# Development of Basic Motor Skills from 3 to 10 Years of Age: Comparison by Sex and Age Range in Chilean Children

**DOI:** 10.3390/children11060715

**Published:** 2024-06-11

**Authors:** Juan Hurtado-Almonacid, Tomás Reyes-Amigo, Rodrigo Yáñez-Sepúlveda, Guillermo Cortés-Roco, Cristian Oñate-Navarrete, Jorge Olivares-Arancibia, Jacqueline Páez-Herrera

**Affiliations:** 1 eFidac Research Group, Physical Education School, Pontificia Universidad Católica de Valparaíso, Valparaíso 2340025, Chile; juan.hurtado@pucv.cl (J.H.-A.); jacqueline.paez@pucv.cl (J.P.-H.); 2 Physical Activity Sciences Observatory (OCAF), Departament of Physical Activity Sciences, Universidad de Playa Ancha, Valparaiso 2360072, Chile; tomas.reyes@upla.cl; 3 Faculty of Education and Social Sciences, Universidad Andres Bello, Viña del Mar 2520000, Chile; rodrigo.yanez.s@unab.cl; 4 School of Education, Sport Coach, Universidad Viña del Mar, Viña del Mar 2572007, Chile; guillermo.cortes@uvm.cl; 5 Department of Therapeutic Processes, Faculty of Health Sciences, Universidad Católica de Temuco, Temuco 4780000, Chile; cristian.onate@uct.cl; 6 AfySE Group, Research in Physical Activity and School Health, School of Physical Education, Faculty of Education, Universidad de las Américas, Santiago 7500975, Chile

**Keywords:** motor development, fundamental movements, motor ageing

## Abstract

Basic motor skills are recognized as fundamental movements that allow children to interact with their environment and are identified as the basic structure on which more complex movements are built. Objective: to identify the level of motor development of children from 3 to 10 years of age according to sex and age group. Methodology. We studied a sample with a total of 328 participants (girls = 170; boys = 158) at preschool (*n* = 154) and school levels (*n* = 174). The ages of the students ranged from 3 to 10 years, with a mean of 5.94 years (±2.53). TGMD2 was applied to identify motor development. Results: boys and girls present low levels of physical activity, with most of them in the very poor, poor, and low-average categories (*n* = 182, 55.5%). Levels of motor development in locomotion, manipulation, and general development by age show significant differences (*p* = 0.000). However, levels of development by sex are not significant in manipulation, locomotion, and gross motor skills tests, respectively (*p* = 0.150, *p* = 0.208, and *p* = 0.210), and in relation to chronological age and motor development age show significant differences (*p* = 0.000).

## 1. Introduction

The infant stage (0 to 6 years) is a period of great relevance for the development of motor skills [1,2]. These can be classified according to the muscle groups involved in the movement and developmental taxonomies. According to developmental taxonomies, basic motor skills are those movements considered structural and the basis of skills that require greater complexity, whose acquisition and development is associated with the influence of structural aspects and a strong anthropometric influence [3,4,5]; they can be classified into non-locomotor stability, locomotor skills, and manipulative skills [1]. They are also recognized as structured movements, following reflex movements and rudimentary movements, in which the acquisition of mature patterns of movement in manipulative, locomotor, and balance behaviors progresses [6]. Hulteen et al. [7] provide a more contemporary classification of motor skills, indicating that these respond to fundamental movement skills, which correspond to movement patterns directed to a specific motor goal and that directly influence the person’s ability to be more physically active. Further, they better reflect the vast range of forms of motion that become increasingly complex and specific, as well as having greater applicability to various environments. These movements are the foundation on which other, even more complex movements are built. Through them, the child progresses in the development and acquisition of mature patterns that allow them to interact with the environment and acquire experiences for coexistence and full participation in the social sphere [7]. Similarly, Hulteen et al. [7] indicate that these fundamental movement skills consistently and permanently support the development of human movement, leading to skilled performance and the development of movement skills that supports and maintains a lifetime of physical activity [8,9]. They demonstrate a cumulative and sequential nature, where the term fundamental restricts the broad range of competencies. The term “foundational movement skills” refers to the fact that there is no predetermined set of “foundational” movement skills, an idea that allows for greater openness to consider other types of skills. Therefore, it is proposed that foundational movement skills encapsulate both movement skills traditionally considered “foundational” as well as other movement skills that are important to promote the development of physical activity over the course of one’s life [7]. These fundamental movement skills are strongly related to childhood development, a stage that has been recognized as a fundamental pillar for the development of social, motor, and cognitive skills. Studies also argue that this stage is critical for human development and learning [10,11,12], as fundamental movement skills play an essential role, since they represent the basic functional unit of movements that are more complex [13,14,15,16,17,18,19,20,21] and that favor the acquisition of mature movement patterns [22,23]. Their nature is mainly ontogenetic, and they are influenced by dynamic interactions with the environment [19,23] in which biological, psychosocial, and environmental factors have an influence [1,13,24,25,26,27]. These fundamental movement skills enable children to successfully cope with the challenges of everyday life, interact with the environment, and adapt to a changing environment [13]. They are relevant because they are recognized as precursors of physical activity and also of sedentary lifestyles [24,28,29]. In addition, children with appropriate learning environments can achieve substantial improvements, as this is a period of maximum sensitivity and vulnerability to environmental influences [25,29,30]; thus, the first life cycle, especially between 2 and 6 years of age, has a strong impact on the development of skills, since it is identified as a sensitive period in motor construction [4,28,29,30,31,32,33] and of influence for later stages [1,34,35]. They show that typically developing children have the potential to be proficient in many skills by the age of 6 years [20]. The development of these fundamental movement skills is characterized by continuous modification during childhood, based on the interplay between neuromuscular maturation, which is primarily genetically controlled, growth, the effects of previous motor experiences, and new motor experiences [35,36]. Development is closely linked to other areas of the human being, for example, it impacts on individual health, cognitive capacity, emotional well-being, and social development, and it has been proven that children with high scores in motor development tests advance more academically [37]. There are differentiated rhythms in levels of motor development, with higher motor performances observed at 3 and 4 years of age [1]; on the other hand, strong correlation coefficients have been found between motor performance and chronological age in preschool children aged 3 to 5 years with typical developments. [38,39].

From the point of view of sex, differences have been found in the basic motor skills of boys and girls [40]. Boys have greater competence in object control skills than girls. On the other hand, no differences in locomotion skills were observed between girls and boys. [3]. Studies consistently indicate that boys are more proficient in object control skills [19,41] and also show higher levels in motor tasks related to object manipulation and control [41,42]; however, in skills related to locomotion and body control, there is no concrete consensus. It is likely that these sex-specific differences in the competence of fundamental movement skills account for the influence of environmental and socio-cultural factors, as well as the level of family support and stimulation received. Considering these differences, there is evidence of low levels of competence in this age group, in which boys and girls do not reach mature stages in fundamental movement skills, as well as indicating that motor competence remains low at the age of 6 to 10 years [17,21].

Previous results have indicated that children in this age group have decreased scores in gross motor coefficients [12,43]. On the other hand, evidence shows that 4.4% of children aged 3 to 6 years have decreased developmental scores, and 8.8% are at risk of delay, and these decreased scores are associated with difficulties in achieving recommended levels of physical activity [1]. On the other hand, evidence indicates that children with higher levels of fundamental movement skills tend to be more active and perform more physical activity than those with poorer skills [44]. Recently, clear evidence has been found that physical activity decreases for each year of a child’s life, starting in early childhood [3,29,30]. This is a complex situation, given that reduced levels of physical activity can jeopardize the development of fundamental movement skills, fitness, and health. In addition, evidence suggests that when motor skills are poor, social status and adiposity levels are some of the factors that most influence the qualitative aspects of movement during childhood [45,46,47]. The age of seven years constitutes a fundamental stage, since it is expected that at this age acceptable levels of quality are reached in fundamental movement skills, a situation that could be favored as participation in motor manifestations of greater specificity increases. For example, through postural control, children can reach and see new parts of their environment; through locomotor skills, it is possible to access new places and expand one’s knowledge of space; through object control skills, it becomes possible to interact with different objects, as well as to increase one’s opportunities for social interaction. Therefore, adequate fundamental movement skills are considered important for the overall development of the child, i.e., their physical, cognitive, and socioemotional development. Given this background, the aim of this study is to identify the motor development of children aged 3 to 10 years, according to age range and sex, as well as to understand the motor age as a function of the chronological age of children.

## 2. Materials and Methods

The method used is based on the quantitative paradigm, with a descriptive, non-experimental, cross-sectional study.

### 2.1. Participants

The sample was obtained non-probabilistically and by convenience, with a total of 328 participants (girls = 170 (51.8%); boys = 158 (48.2%)) of preschool (154 students, 47%) and school (174 students, 53%) educational levels from the region of Valparaíso, Chile. Students’ ages ranged from 3 to 10 years, with a mean age of 5.94 years (±2.53); the mean age of females was 5.9 years, and the mean age of males was 5.8 years.

### 2.2. Procedure

The protocols were applied considering the ethical principles for research with human beings proposed by the Declaration of Helsinki (World Medical Association) in addition to the above. This research project was approved by the research department of the Pontificia Universidad Católica de Valparaíso through the Scientific and Bioethical Ethical Committee (BIOEPUCV-H-456-2021). On the other hand, the school authorities of the educational establishments were asked for authorization to carry out the research project. Once the research project was approved by the authorities, an informed consent form was sent to the parents and/or guardians, indicating the objectives and scope of the study in order to authorize their child’s participation. Then, during physical education classes, the evaluation instrument was applied, starting with locomotion tests and then moving on to manipulation tests. The test was administered by 10 researchers. This included wo physical education teachers responsible for the project and with vast experience in the application of the TGMD-2, and a total of eight students with careers in physical education pedagogy, assigned to the eFidac research laboratory. The latter were trained for a period of 2 weeks. The first one was theoretical, where they learned about the test, the achievement indicators, and the procedure used to establish the classification obtained from the scores. A second training period was of a practical type, where the evaluators were able to perform the corresponding tests, according to the established protocols, and evaluate a smaller group of children, who attended the school.

For the application of the TGMD-2, two teams were formed, and each was composed of 1 academic and 4 students, who were responsible for each dimension (one team with the object control tests, and one team with the body control tests).

It should be noted that the measurements were taken after the end of the COVID-19 pandemic, so that all children participating in this study experienced total confinement as a result of the pandemic. After the application, a report was given to the educational establishments incorporating some recommendations according to their results.

### 2.3. Instrument

The TGMD-2 instrument was used to assess motor competence in children, and is the most widely used instrument in the world to measure motor development [46], with validity indices of 0.93 for language clarity and 0.91 for relevance [4]. The purpose of this instrument is to evaluate the motor development of children aged 3 to 10 years and 11 months, classifying motor behaviors in seven categories: very deficient, deficient, mediocre, mediocre, superior, and very superior. The test identifies these categories of motor development according to sex and years of age in months, considering tests of locomotion motor skills, object control skills, and total motor development. In locomotion skills, tests of running, galloping, jumping on one foot, jumping horizontally on two feet, running over an obstacle, and lateral displacement are evaluated, while, in object control skills, tests of catching, bouncing, rolling, hitting a ball with the foot, hitting a ball with a bat, and throwing are evaluated. This is intended to assess three dimensions, locomotion, manipulation and gross motor development.

The evaluation was performed on a flat surface, which was an obstacle-free space, and participants were asked to attend the evaluation in comfortable clothing. The locomotion and object control tests were carried out individually. To assess locomotion skills, the tests were organized into stations in the following order: running, galloping, jumping, galloping and jumping on one foot, jumping horizontally on two feet, running over an obstacle, and lateral displacement. The object control tests were also distributed in successive stations in the following order: catching, bouncing, rolling, hitting a ball with the foot, hitting a ball with a bat, and throwing [48,49].

Each motor skill is evaluated by means of three to four performance indicators, in which a score of 1 is recorded for a correct performance and 0 otherwise. Once the test is applied, the two attempts per test of the two subtests are added, and then the scores obtained are analyzed with the conversion table according to the age in months of the child, which provides a score called a standard score that describes a Gross Motor Quotient, where it ends with the description of the range in seven categories: very superior > 130, superior 121–130, above average 111–112, average 90–110, below average 80–89, poor 70, 79, and very poor < 70 [32,49,50]. The test from the standard score for locomotion and manipulation provides information on the motor age attained, which is obtained according to the results of the different tests of both skills.

### 2.4. Data Analysis Technique

The data were analyzed using IBM SPSS Statistics 25 software (New York, NY, USA). First, a descriptive analysis was used based on relative and absolute frequencies, with the purpose of grouping the participants according to the level of motor development achieved. This allowed us to first group girls and boys according to their level of motor development, and then we grouped the participants according to age range and level of motor development, both for object control and body control. The Kolmogorov–Smirnov test (*n* > 50) was used to determine the normal distribution of the data. From the ordinal and nominal categorical variables, the non-parametric Chi Square test was applied to test the statistical significance with a confidence level of 95% and a 5% error rate.

Statistical significance was determined at a *p*-value equivalent to *p* < 0.005.

## 3. Results

The main results are presented below, where Table 1 shows the participants according to sex, educational levels, average age, and four age range classifications from 3 to 10 years old. In order to analyze the information, the categories of 3–4 years old, with 64% of the participants, 5–6 years old, with 14%, 7–8 years old, with 32.7%, and finally, children aged 9–10 years old, with 26.4%. Similarly, the classifications for motor development are presented, where they are grouped into three categories to analyze the information: Category 1 (very poor, poor, and low average), where most of the children were found, comprising 74.7% of the total sample; in relation to the sexes, 98 girls were recorded, corresponding to 57.7%, and 84 boys were recorded, corresponding to 53.2%; Category 2 (average motor development level), with a total of 36.6% of the total sample, where there were 61 girls (35.9%) and 57 boys (36%) recorded; and Category 3 (average, above average, and very above average), where only 8.53% of all students were found, comprising 11 girls (6.4%) and 17 boys (10.8%).

Table 2 shows the general motor development, together with locomotion and manipulation, achieved according to the four age categories. In relation to manipulation, it can be observed that 145 students are in the category comprising very poor, poor, and low average, representing 60.9% of the total sample, where the age category of 7–8 years presents the highest number of children (61). Then, in Category 2 (average), there are 153 students, representing 64.2% of the total sample, where there is the highest number of students of 3–4 years old, totaling 99 students. Then, in the category comprising above average, very superior, and superior, there are only 30 students, with a total of 12.6%, where no children aged 5–6 and 7–8 years achieved this level of development.

In relation to the locomotive ability, there are 153 students in the category, representing 46.6% of the total sample, while the category of average development includes 152 students, representing 46.3% of the sample, where the age range of 3–4 years is the most prevalent in children in this category, representing 64.7% of the sample. The category including above average, superior, and very superior classifications includes 23 students, representing 7% of the total sample, where again the ages 5–6, 7–8, and 9–10 years have the lowest number of students.

In relation to general motor development, 182 students (55.5% of the total sample) are in the poor, very poor, and low-average categories, while 118 students (35.9%) are in the average category, and only 28 students (8.6%) are in the above average, superior, and very superior categories. The table also shows significant differences, (*p* = 0.000) with *p* < 0.005, showing significant differences in manipulation, locomotion, and general motor skills.

Table 3 shows the level of motor development according to the sex of the participating children, where, in manipulative skills, 145 students are in the very poor, poor, and medium-low categories (44.2%), where boys have better results in the three categories of motor development levels, with no significant differences (*p* = 0.150), with *p* < 0.005. In relation to the ability for locomotion, 153 students are in the category of very poor, poor, and low average, with 46.6% of the sample, where the girls present better results in the category of average and the boys in the above average, superior, and very superior categories, but with these differences not being significant (*p* = 0.208). Finally, we present the general motor development, where 182 students score in the very poor, poor, and low-average categories (55.4%), with a greater presence of girls in this category. In relation to the average category, once again, the girls present a greater number, which is modified in the last category of above average, very superior, and superior, where the boys present better results, but with these not being significant differences (*p* = 0.210).

Finally, Table 4 shows the comparison of the averages according to the chronological ages of the boys and girls in relation to motor age according to the TGMD2, where both the age of locomotion and manipulation are lower in relation to their chronological age, and with this difference being significant (*p* = 0.005).

## 4. Discussion

Regarding the level of motor development in the children, the findings indicate that the scores are lower for locomotion, manipulation, and general motor development skills, where most of the children are in the very poor, poor, and low-average categories [3,21]. These results are related to the analysis of the studies of other research works, such as those presented by Ancheta-Arrabal [12] where decreased results in locomotion, manipulation and total motor development tests were also reported, which indicate that there are previous studies that used the TGMD-2. Poor values were found for the gross motor coefficients, such as those of Dobell et al. [51], those of a study conducted with 267 Canadian preschoolers aged 5 ± 0.9 years, and a study by Morales et al. [52], with 284 Brazilians aged 3–6 years, with similar results. These results also coincide with a study by Lawson et al. [15], where 219 children (111 boys and 108 girls) aged 7 to 10 years were evaluated under the TGMD2, where they presented low levels of general motor development and where no significant differences by sex were found.

In a research study conducted with 136 preschoolers (70 girls and 66 boys) between 4 and 5 years of age, it was found that both sexes had average motor developments and only a very low percentage of children placed in the above-average and above-average levels, and no preschoolers were placed in the above-average and above-average levels, nor were they placed in the very superior category [53]. This is repeated in a study of 60 schoolchildren aged 9 to 12 years. While the results of this study indicated that the developmental levels of the schoolchildren were lower than in the results of this study, the levels of the schoolchildren were below average, with 46% of the sample in the general classification of the TGMD-2 test [54].

From the sex difference, our research did not find significant differences between developmental levels in locomotion, manipulation, and general development. Similarly, ref. [12] indicates that studies that have compared male and female preschoolers found no differences in gross motor coefficients [28,55]. They also point out that this same result was obtained with a sample of 71 Europeans (5.58 ± 0.27 years) in a study by Foulkes et al. [55,56,57].

There is evidence that, when relating the level of the motor development of Chilean children to the level of physical activity of their parents, it is possible that the level of physical activity of their parents may be related to the level of their own motor development. It was found that girls obtained lower scores in locomotion and manipulation tests. Also, a higher percentage of girls were found in the very poor, poor, and low-average categories in relation to their male peers. These results coincide with those presented in this study [55].

Regardless of the sex of the participating child, it is possible to observe that, to a greater extent, they are located in the very poor, poor, low-average, and average levels of motor development. These results coincide with a study carried out in Chile, the purpose of which was to relate the level of motor development to the level of physical activity of children between 4 and 6 years of age participating in the state program “Crecer en Movimiento” (Growing in Movement). The results of this study indicated that children in the lower age ranges presented the lowest results in basic motor skills of locomotion and object control [56]. This situation can be explained by the fact that, at a younger age, children are still in an initial phase in the development of their motor skills, since their levels of experience and systematization of practice are still low [56,57].

In relation to differences in skill type, the study found that boys performed better in manipulation skills and girls performed better in locomotion skills. This was similar to Hardy et al. [31], in a study with 425 preschool children in Sydney, where boys performed better in object control and girls performed better in locomotion skills. This situation coincides with the above in a study by Martins et al. [58] who, when evaluating the motor development of 6241 Brazilian children aged 3 to 5 years, concluded that girls presented better performance in locomotion tests mainly between 57 and 59 months, as well as at 66 and 68 months. Boys outperformed girls in ball skills tests in all age groups. These results are also consistent with boys and girls in China, where girls performed better in locomotion tests and boys scored better in ball tests. He et al. [59] indicate that these differences are mainly due to sport preferences, as well as to environmental and social factors, where boys receive more support to participate in physical activity and sports, both at school and in their own community.

From the analysis of the differences between types of skills, the authors of [12] point out that in a study [55] with samples of 168 English preschoolers, a study [28] with 425 Australian children aged 4 years, and a study with 339 Americans aged 3–5 years [55], the scores in locomotor skills were higher than those of manipulative skills.

The study found significant differences in relation to the chronological age and the motor age of the children, with the motor age being lower than the chronological age. This coincides with the results presented by the authors of [35], in an intervention study to improve motor development, which showed that 93% of the children evaluated had a lower level of motor development than the chronological age. However, there is evidence that indicates that the higher the chronological age, the higher the motor age, a situation that is explained by the fact that as motor performance increases [60], there is also a greater maturation of the central nervous system [58,59,61].

Another important element of this research work is that information was collected after the confinement resulting from the COVID-19 pandemic; in this sense, the children evaluated lived part of their initial school years in confinement. In this regard, Cárcamo-Oyarzún et al. [62,63] indicated that the COVID-19 pandemic has subsequent effects, such as a decrease in the development of basic motor skills, which would have a direct impact on the physical inactivity of schoolchildren [63,64].

Merce et al. [65], upon investigating the impact of the COVID-19 pandemic on the physical activity levels of Portuguese schoolchildren, indicated that they were more physically active before the pandemic. This issue was explained by the low demands of movement that children had at the time of online classes. However, this situation is not new, as there are results that indicate that there was a considerable reduction in physical activity, regardless of the age of the individuals, but this impact is not statistically significant [66]. Promoting spaces for physical activity and sport practice seems fundamental in childhood, since those children who engage in physical activity and sport during this stage tend to remain more active in adulthood. Thus, the development of fundamental movement skills will act as an enhancer of physical activity levels [67,68,69].

Among the limitations of this study are the small number of participating children, and, given that the sample is not representative of the population, it is not possible to extrapolate the results obtained. It should also be noted that another important limitation of this study is the failure to incorporate other analyses into the results, such as the socioeconomic level of the participants. Finally, another important limitation is, from an ecological approach, that it would be interesting to investigate the influence of the fathers, mothers, or guardians responsible for the motor development of these children, as well as the influence of the school context.

## 5. Conclusions

In relation to the level of motor development of the children, these are found to be lower for the skills of locomotion, manipulation, and general motor development, where most of the children are in the very poor, poor, and low-average categories. From the sex difference, our research did not find significant differences between developmental levels in locomotion, manipulation, and general development. Finally, this study found significant differences in relation to the chronological age and the motor age of the children, with the motor age being lower than the chronological age.

Among the main projections, it has been determined crucial to advance in interventions in the school context, with a focus on the development of skills that contribute to the state of motor skills in childhood.

Within the future lines of research, we plan to carry out intervention programs based on motor development that consider the teaching styles of teachers and the classification system proposed by Ahmadi et al. [70]. A motor development intervention program, where the basic psychological needs of children are addressed using effective teaching strategies, would promote the learning of fundamental movement skills in a profound way.

## Figures and Tables

**Table 1:** Characterization of participants according to sex.

School Levels	Female *n* = 170 (51.8%)	Male *n* = 158 (48.2%)	Total *n* = 328
Preschool	77 (50%)	77 (50%)	154
Schoolchildren	93 (53.4%)	81 (46.6%)	174
Average Age (ages)	5.9	5.8	5.94
Age range			
3–4 years.	76 (49.6%)	77 (50.4%)	153
5–6 years.	20 (58.8%)	14 (41.2%)	34
7–8 years.	43 (55.2%)	35 (44.8%)	78
9–10 years.	31 (49.2%)	32 (50.7%)	63
Levels of motor development (general)			
Category 1			
Very Poor	32 (62.7%)	19 (37.3%)	51
Poor	45 (57%)	34 (43%)	79
Low average	21 (40.3%)	31 (59.6%)	52
Category 2			
Average	61 (51.6%)	57 (48.4%)	118
Category 3			
Above average	6 (40%)	9 (60%)	15
Above	5 (41.7%)	7 (58.3%)	12
Very Superior	0	1 (100%)	1

**Table 2:** Levels of motor development according to age.

Levels of Development		3–4 Years *n* (%)	5–6 Years % (*n*)	7–8 Years % (*n*)	9–10 Years % (*n*)	*p* Value ¹
Manipulation	Very Poor, Poor, Low average	25 (16.4%)	24 (70.5%)	61(78.3%)	35 (55.6%)	0.000
	Average	99 (64.7%)	10 (29.5%)	17 (21.7%)	27 (42.9%)	
	Above average, Superior, Very Superior	29 (18.9%)	0	0	1 (1.5%)	
Locomotion	Very Poor, Poor, Low Average	37 (24.1%)	19 (55.8%)	53 (67.9%)	44 (69.8%)	0.000
	Average	98 (64%)	15 (44.1%)	23 (29.4%)	16(25.3%)	
	Above Average, Superior, Very Superior	18 (11.7%)	0	2 (2.56%)	3(4.76%)	
Gross Motor	Very Poor, Poor, Low Average	35 (22.8%)	30 (88.3%)	69 (88.4%)	48 (76.2%)	0.000
	Average	93 (60.8%)	4 (11.7%)	9 (11.6%)	12 (19.0%)	
	Above Average, Superior, Very Superior	25 (16.4%)	0	0	3 (4.8%)	

^1^ Chi Square test: *p*-value < 0.05.

**Table 3:** Developmental levels of locomotion, manipulation, and gross motor skills, according to sex.

Levels of Development		Female *n* (%)	Male *n* (%)	*p* Value ¹
Manipulation	Very Poor, Poor, Low Average	81 (47.7%)	64 (40.5%)	0.50
	Average	75 (44.1%)	78 (49.4%)	
	Above Average, Superior, Very Superior	14 (8.2%)	16 (10.1%)	
Locomotion	Very Poor, Poor, Low Average	75 (44.1%)	78 (49.4%)	0.208
	Average	86 (50.5%)	66 (41.8%)	
	Above Average, Superior, Very Superior	9 (5.29%)	14 (8.8%)	
Gross Motor	Very Poor, Poor, Low Average	98 (57.7%)	84 (53.2%)	0.210
	Average	61 (35.8%)	57 (36.0%)	
	Above Average, Superior, Very Superior	11 (6.5%)	17 (10.8%)	

^1^: Chi Square test: *p*-value < 0.05.

**Table 4:** Averages according to the chronological ages of the boys and girls in relation to motor ages according to the TGMD2.

Chronological Age/Motor Age	TOTAL *n* = 328	*p* Value ¹
Average age (ages)	5.94	0.000
Locomotion motor age	4.17	
Manipulation motor age	4.49	

^1^ Chi Square test: *p*-value < 0.05.

## Data Availability

The original contributions presented in the study are included in the article, further inquiries can be directed to the corresponding author.
